# Informing a Clinical Pathway for Acute Knee Injuries: Survey Insights From Rural Clinicians

**DOI:** 10.1111/ajr.70086

**Published:** 2025-08-27

**Authors:** Tom Molloy, Benjamin Gompels, Matthew Dowsett, Stephen McDonnell

**Affiliations:** ^1^ Faculty of Medicine University of Queensland, Mayne Medical School Herston Queensland Australia; ^2^ Division of Trauma and Orthopaedic Surgery University of Cambridge, Addenbrooke's Hospital Cambridge UK

**Keywords:** diagnosis, injury, knee, pathway, rural

## Abstract

**Introduction:**

Soft tissue knee injuries (STKIs) pose a significant healthcare challenge, particularly in rural settings with limited access to imaging and specialist consultation. This study aimed to evaluate current practices and challenges in diagnosing acute knee injuries in a rural setting and presented a pathway tailored for rural healthcare settings to improve diagnostic confidence, optimise imaging use, and streamline patient management.

**Methods:**

A survey‐based study was conducted among seventeen medical officers in six rural medical centres across New South Wales and Queensland. The survey involved a structured questionnaire on current practices and challenges. A structured rural pathway for acute knee injury was presented, and feedback was reported.

**Results:**

Most clinicians assessed knee injuries weekly, showing variable confidence in their assessments and special tests. Key barriers identified included limited access to imaging, lack of specialist consultation, and diagnostic uncertainty, which led to increased referrals. The proposed pathway was rated highly intuitive, aligned with clinical guidelines, and was expected to streamline management.

**Conclusion:**

The proposed pathway has clinical support and the potential to enhance knee injury management in rural settings by improving diagnostic accuracy, offering pathways aligned with the risk of injury, and promoting timely specialist care. Further research is necessary to assess long‐term clinical outcomes and pathway integration across allied health services and rural healthcare facilities.

**Summary**

- Clinicians' confidence varies in assessing acute knee injury, with challenges in pain, effusion, and subsequent imaging and referral decisions.
- Rural settings face significant barriers to assessment of STKIs due to limited imaging, specialist access, and training gaps.
- The proposed risk stratification and management pathway is well‐received and considered useful by clinicians.

## 1 Introduction

Soft tissue knee injuries (STKIs) represent a significant healthcare burden with an increasing incidence and the potential for substantial physical and mental health consequences [1]. Ligamentous and meniscal knee injuries are associated with the development of early‐onset osteoarthritis, where chronic pain, reduced mobility, and limitations in daily activities can severely affect quality of life [2]. Additionally, the psychological burden of living with chronic pain and disability may lead to depression, anxiety, and social isolation, potentially worsening treatment outcomes [3]. Furthermore, patients with limited healthcare access can have even poorer outcomes [4]. As such, streamlining diagnostic and management pathways to improve the healthcare outcomes for vulnerable populations has been highlighted as a global research priority [5]. Rural populations are a key consideration in this area, often from lower socioeconomic status (SES) backgrounds and with limited access to healthcare [6]. It has also been reported that lower SES is associated with higher rates of osteoarthritis. Given the established link between intra‐articular injury and post‐traumatic osteoarthritis, timely and accurate diagnosis of STKIs in rural, low‐SES settings should be a priority to reduce long‐term complications and improve patient outcomes [7, 8].

There is a range of barriers to achieving an accurate and timely diagnosis of STKIs. In the acute setting, accurate diagnosis is complicated by the issues of pain, guarding and effusion [9]. These issues make it difficult for a clinician to conduct a complete clinical assessment that includes the appropriate application and interpretation of special tests [10]. This is compounded by the fact that diagnosis depends on the clinician's proficiencies, where experience and training directly correlate to the accuracy of diagnosis [11]. Furthermore, diagnostic imaging is heavily relied on, as magnetic resonance imaging (MRI) is the gold standard of diagnosis [10]. This poses significant problems of affordability and accessibility to the gold standard diagnosis across all healthcare settings.

In the rural context, all these issues may be exacerbated, and diagnosis can become increasingly complex. In rural settings, many patients with acute STKIs present to primary care clinics, small emergency departments, or remote healthcare facilities with limited access to specialist consultation and imaging, such as out‐of‐hours radiographs and MRI. As rural clinicians may have less experience in musculoskeletal assessment, conducting a clinically accurate evaluation can become even more difficult, leading to potentially increased diagnostic uncertainty. The lack of on‐site or local orthopaedic or sports medicine specialists for consultation may result in delayed or unnecessary referrals. An increase in referrals will inevitably create inefficiencies in the diagnostic pathway and delay the necessary treatments.

Regarding diagnostic imaging, MRI is often unavailable in rural hospitals and clinics and is also unaffordable by rural populations who frequently live in low to medium SES communities [12]. Where MRI is unavailable, and X‐ray and ultrasound do not provide any diagnostic information, rural populations are left with limited imaging options. Subsequently, in rural settings, as special clinical tests offer limited accuracy and diagnostic imaging is unattainable, there is a significant gap in achieving an accurate diagnosis for STKIs and timely management decisions.

As such, supporting the accurate diagnosis of STKIs is crucial to ensure prompt treatment and avoid missed injuries. Without clear decision‐making pathways, healthcare providers may struggle to determine whether an injury warrants referral, leading to inconsistent management, overuse of imaging, and unnecessary patient transfers. In these settings, clinical decision‐making pathways are useful, where evidence‐based guidance is utilised to instil confidence in healthcare workers [13]. Specifically, clinical imaging and referral guidelines aim to optimise the use of radiography and consults, reducing unnecessary imaging and consults while ensuring that significant injuries are not missed [14].

Clinical decision tools have been shown to improve patient outcomes, reduce medical errors and enhance clinical efficiency across various medical fields. On an individual basis, they provide a standardised approach for clinicians with evidence‐based recommendations at the point of care [15]. The effectiveness of these tools has provided the framework for this research, where it is suggested that the combination of multiple clinical decision tools may provide a clear pathway for clinicians to follow to promote confidence in clinical decision making. In this context, a structured acute knee injury pathway combining bony and soft tissue injury tools may improve decision‐making, optimise imaging use, and provide confidence in discharging patients safely. Current imaging guidelines for bony injuries of the knee include the Ottawa Knee Rules and the Pittsburgh Knee Rules [16, 17]. These evidence‐based rules guide clinicians on when to order X‐rays for acute knee injuries with high sensitivity and specificity [18]. Their effectiveness has been robustly demonstrated where the application of the rules has led to a 26% to 31% reduction in imaging while maintaining 100% sensitivity [19]. For soft tissue knee injuries, the Cambridge Knee Injury Tool (CamKIT) is an example of a non‐invasive risk stratification tool that could guide healthcare providers in identifying patients at low, medium, and high risk of significant knee pathology [20]. This simplified tool also demonstrates 100% sensitivity and, when combined with more detailed and thorough STKI management guidelines, could also aid in reducing unnecessary imaging and referrals [21].

In justifying the selection of these two tools, the Ottawa Knee Rules are internationally recognised and validated as a screening tool for bony injury. The Cambridge Knee Injury Tool (CamKIT) is the only available screening tool that assesses risk across all ligamentous and meniscal structures. Other tools are limited to anterior cruciate ligament injury and do not provide utility across the range of soft tissue injuries [22, 23]. The Ottawa Rules and CamKIT were also selected due to their evidence‐based development, cohort validation, and simplicity, as they require no special tests and no diagnostic imaging. These attributes make them uniquely well‐suited to primary care settings, particularly in rural areas, where assessment and diagnostics are not readily available.

**FIGURE 1:**
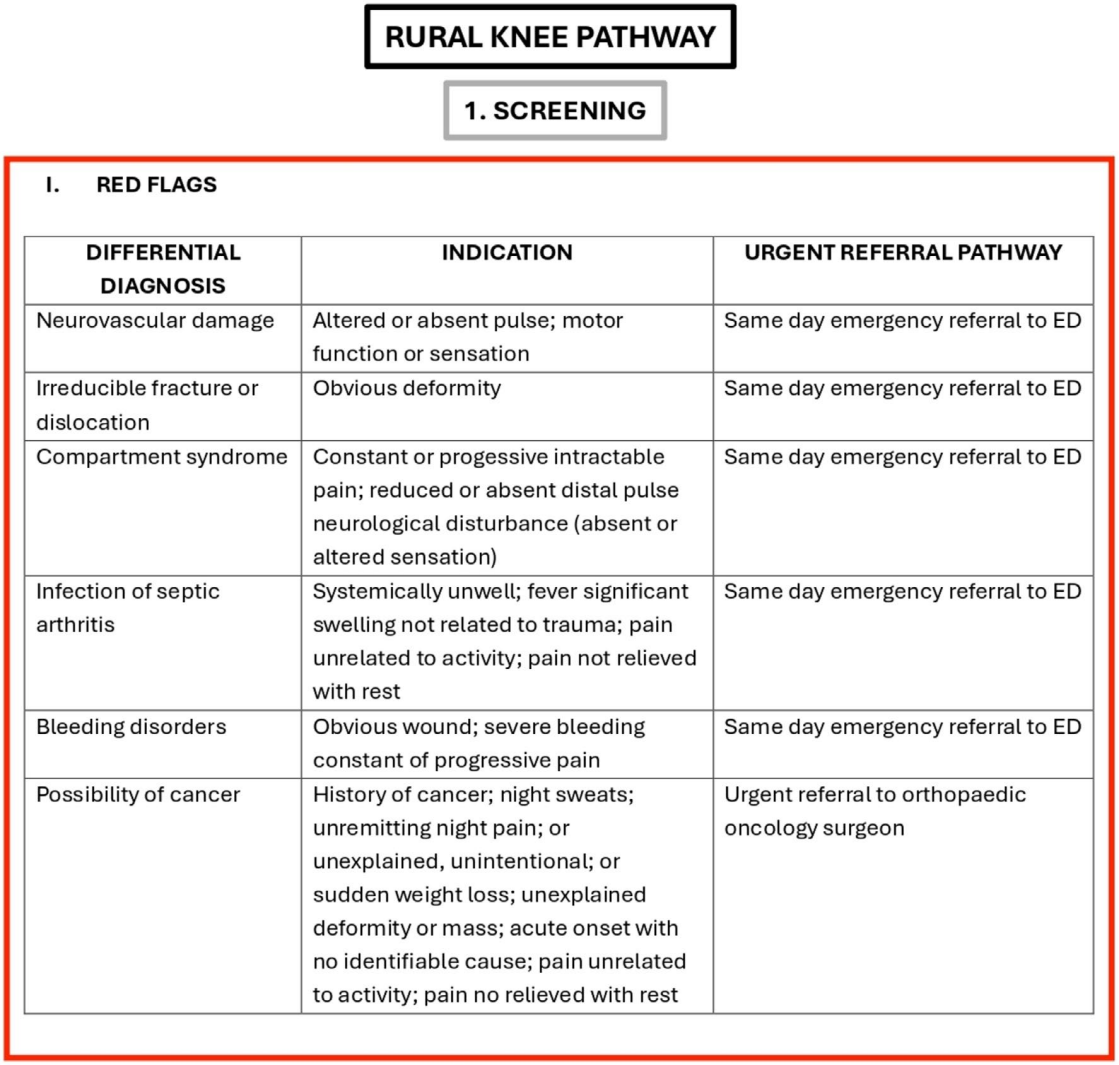
Red flag screening.

**FIGURE 2:**
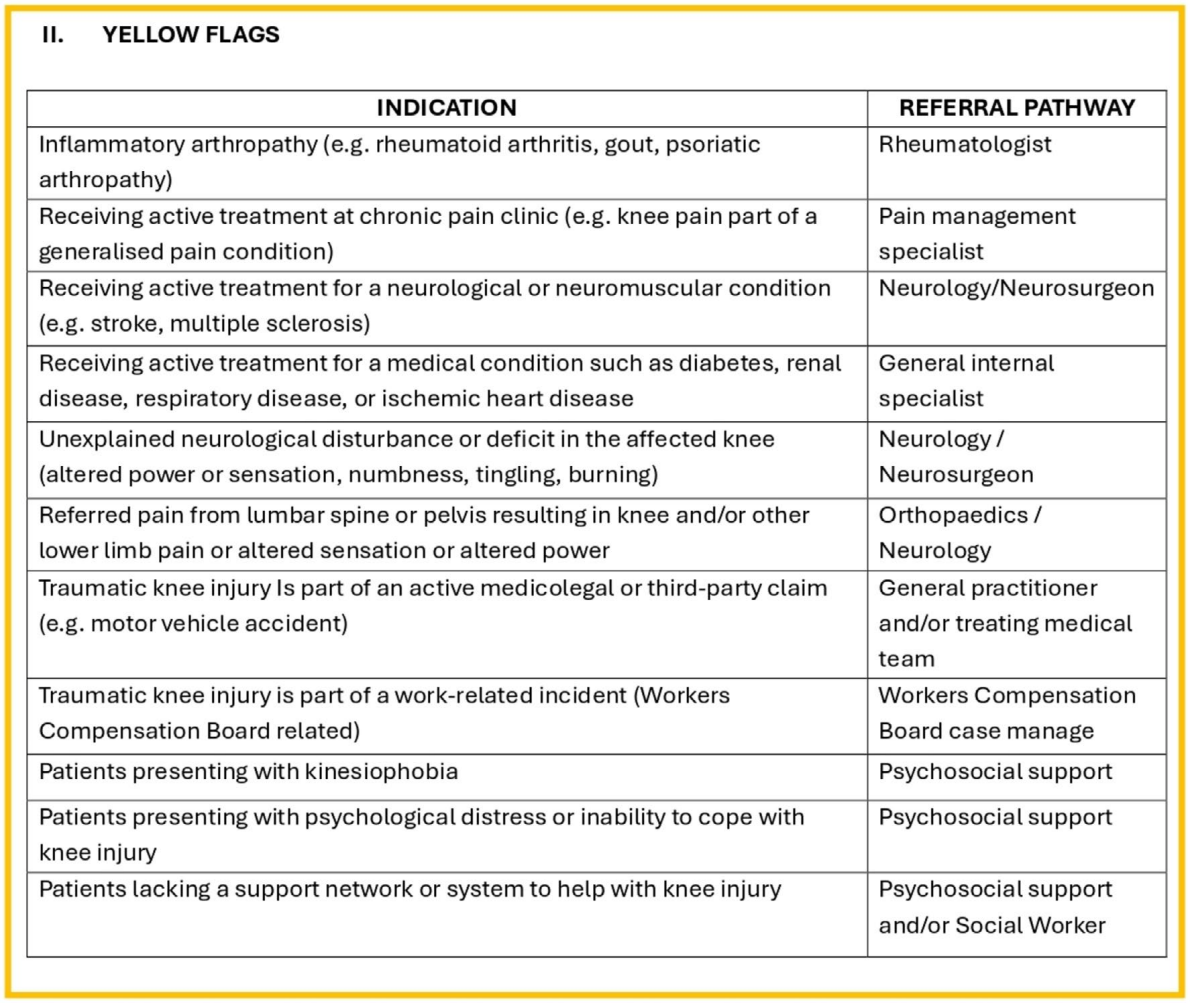
Yellow flag screening.

**FIGURE 3:**
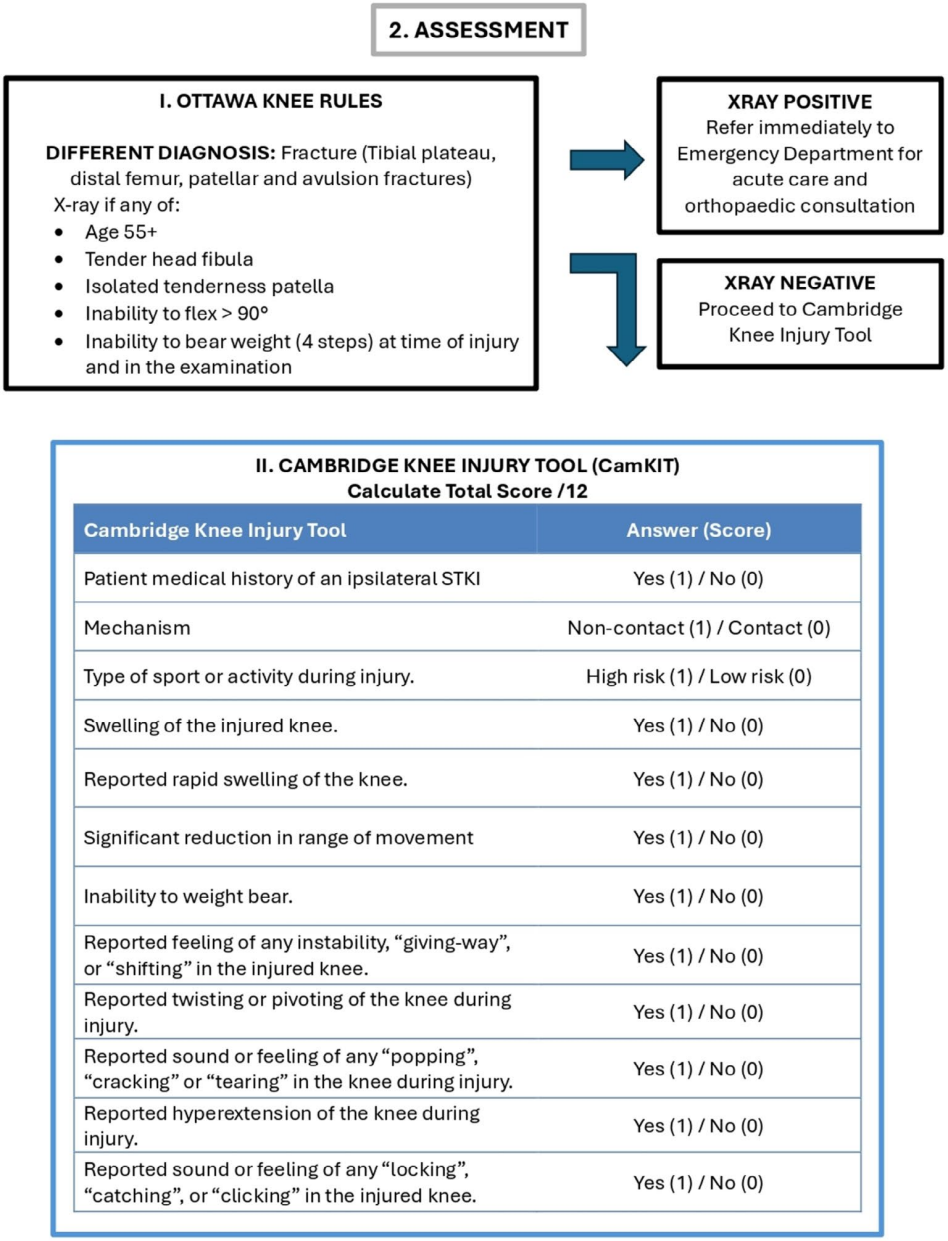
The Ottawa Knee Rules for bony injuries and the Cambridge Knee Injury Tool for soft tissue injuries.

**FIGURE 4:**
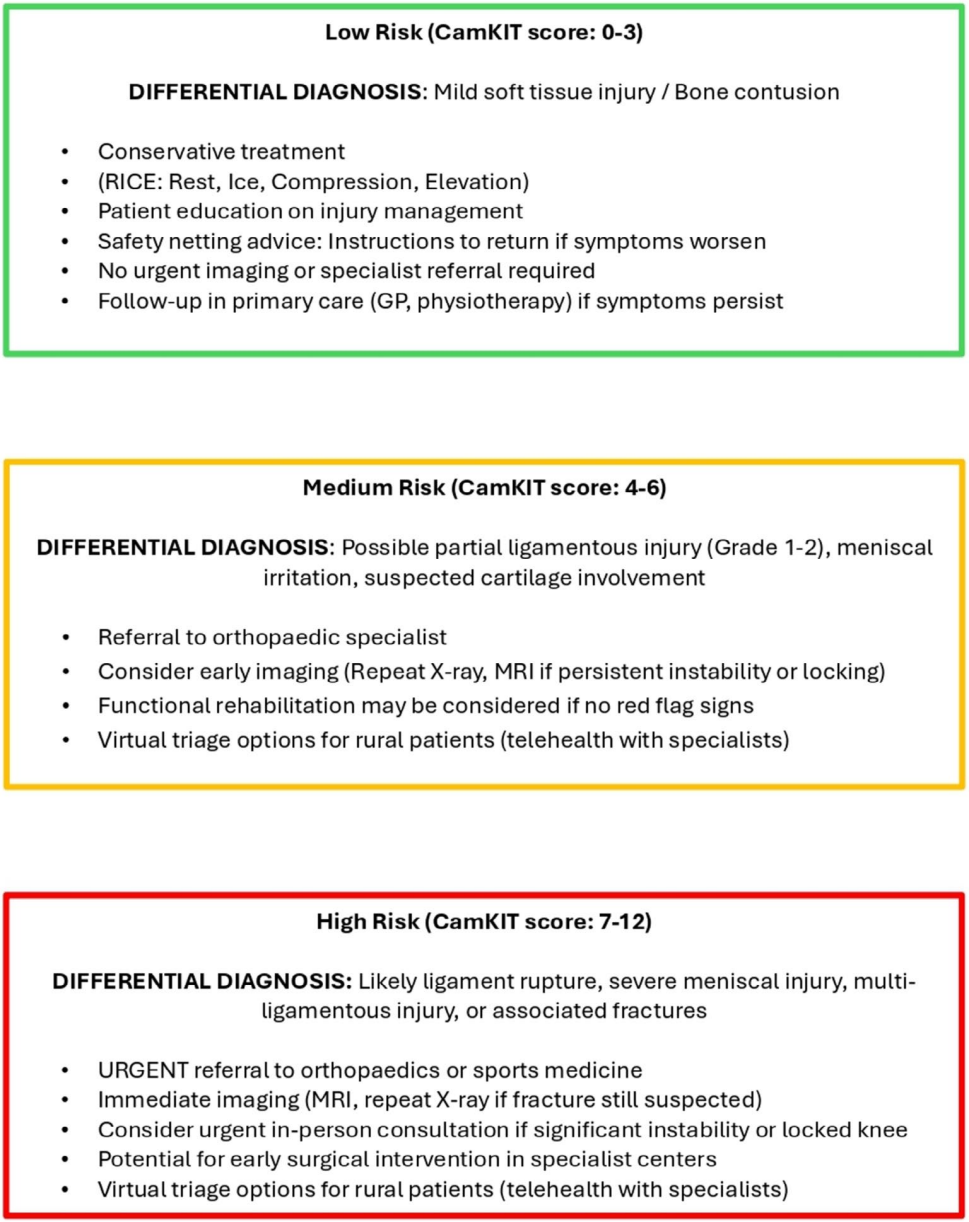
Risk stratification for management and referral pathways for soft tissue injuries.

The study aims to explore the rural clinical perspectives of an acute knee injury pathway that integrates clinical assessment with history and examination, considers red (Figure 1) and yellow flags (Figure 2), and incorporates both the Ottawa Knee Rules and the Cambridge Knee Injury Tool (Figure 3) to guide healthcare decision‐making (Figure 4). Focusing on the perspectives of rural clinicians allows this research to explore the insights of clinicians who experience significant challenges and barriers in resource‐stretched primary care settings, which is a crucial step in informing the development of this pathway. The objectives of the pathway include standardising assessment using clinical risk stratification tools, guiding imaging decisions to ensure the appropriate use of radiographs and MRI based on validated criteria, improving patient flow by safely managing low‐risk cases in primary care, prioritising high‐risk cases for urgent referral (Figure 4), and supporting rural clinicians by integrating telemedicine and virtual triage options for specialist input when needed.

By implementing a structured acute knee injury pathway for bony and soft tissue injuries, rural healthcare providers may be able to confidently assess and discharge patients, reducing unnecessary imaging and referrals while ensuring timely specialist care for those who need it most. This pathway offers a non‐specialist‐accessible framework that has the potential to be applied in pre‐hospital and emergency department settings, where comprehensive examination, imaging, or specialist consultation is not immediately available.

## 2 Methodology

A survey‐based study was conducted among seventeen Medical Officers across 6 rural medical centres, including Alice Springs Hospital, Charleville Hospital, Chinchilla Hospital, Orange Base Hospital, Roma Hospital, and Wagga Wagga Hospital.

A structured questionnaire included a mix of Likert‐scale questions, multiple‐choice options, and open‐ended responses. Part One of the survey focused on assessing perceived competency in knee assessment, awareness of clinical guidelines, use of clinical decision tools for imaging and referral, barriers to accurate diagnosis, and opportunities to develop a structured acute knee pathway to improve patient management. An Acute Knee Injury Pathway was presented to the same group of rural Medical Officers. In Part Two, participants were asked to rate the pathway based on alignment with existing clinical guidelines, perceived utility in improving diagnostic confidence and patient management, ease of use, and feasibility of implementation in their rural hospital setting. The survey is accessible in Appendix S1.

This survey consisted primarily of closed‐ended questions, which were analysed descriptively.

For the open‐ended responses, we provided text entry options for specific questions that may involve options not presented. Previous thematic analysis had been conducted comprehensively in the area of STKIs, and as such, the scope of the rural pathway was therefore based on rigorous prior expert consensus‐building research [5, 24]. The closed nature of the survey precluded the emergence of new qualitative themes, as would be expected in open questioning. Text entry boxes were available at the end of the survey for any additional thoughts and opinions on the topic.

## 3 Results

**FIGURE 5:**
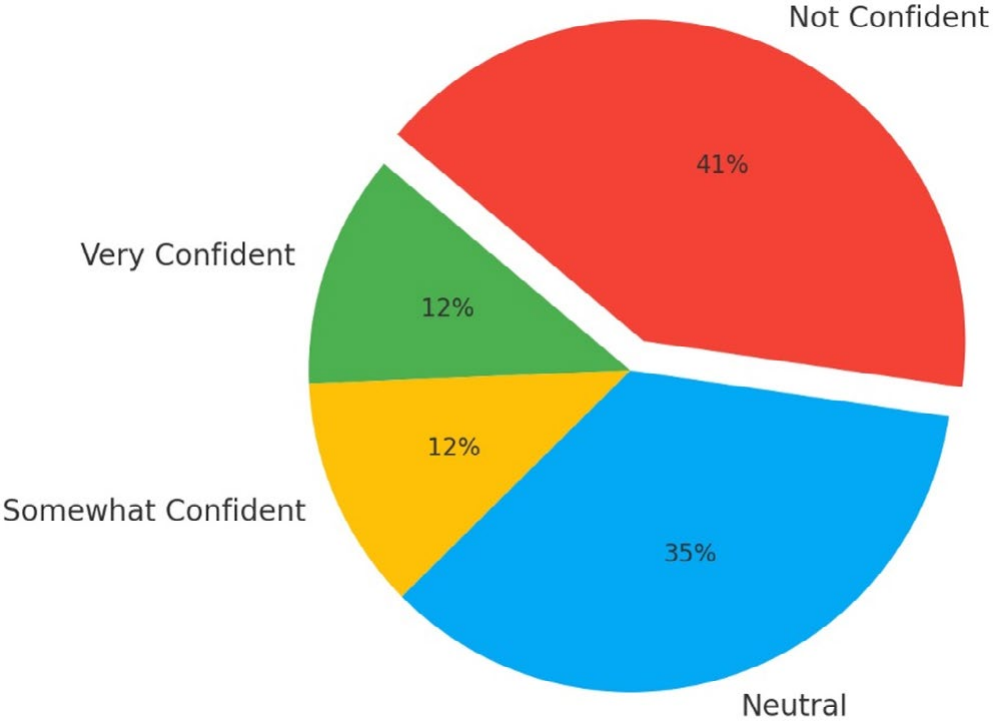
A pie chart illustrating the confidence in performing special tests during knee examinations.

Seventeen clinicians participated in the survey, showcasing a range of clinical experience: 24% (*n* = 4) had over 11 years of experience, 6% (*n* = 1) had between 6 and 10 years, 47% (*n* = 8) had 2 to 5 years, and 24% (*n* = 4) had less than 2 years. Regarding clinical exposure, 59% (*n* = 10) reported assessing acute knee injuries weekly, 24% (*n* = 4) monthly, 12% (*n* = 2) rarely, and 6% (*n* = 1) daily. When asked about their confidence in assessing acute knee injuries, 18% (*n* = 3) described themselves as very confident, 29% (*n* = 5) as somewhat confident, 18% (*n* = 3) as neutral, and 35% (*n* = 6) as not confident. None of the clinicians reported feeling very unconfident. Regarding their ability to perform special tests, only 12% (*n* = 2) felt very confident, 12% (*n* = 2) were somewhat confident, 35% (*n* = 6) were neutral, and 41% (*n* = 7) were not confident (Figure 5). The most frequently cited challenges in knee assessment included pain and guarding during assessment (53%, *n* = 9), joint effusion (53%, *n* = 9), the need for imaging (29%, *n* = 5), and the need for specialist referral (59%, *n* = 10).

Clinicians identified several barriers to effective knee assessment in rural settings. The most common issues were limited access to diagnostic imaging, such as X‐ray or MRI (77%, *n* = 13), lack of access to specialist consultations (47%, *n* = 8), challenges in arranging follow‐up care (59%, *n* = 10), limited training or confidence in knee assessments (41%, *n* = 7), patient reluctance to travel for imaging or consultations (47%, *n* = 8), and concerns about MRI costs (12%, *n* = 2). When asked about referral practices, 35% (*n* = 6) indicated they sometimes feel pressured to refer due to diagnostic uncertainty, 53% (*n* = 9) rarely feel pressured, and 12% (*n* = 2) often feel pressured. Regarding current clinical pathways for acute knee injuries, 47% (*n* = 8) reported that no clear pathway existed, 29% (*n* = 5) found them somewhat effective, and 24% (*n* = 4) rated them as ineffective. None rated their pathways as very effective.

Familiarity with imaging guidelines varied. Only 29% (*n* = 5) were somewhat familiar with the Ottawa Knee Rules but did not consistently apply them; 6% (*n* = 1) were aware but rarely used them, while 65% (*n* = 11) were unfamiliar. For the Pittsburgh Knee Rules, only 6% (*n* = 1) were aware but rarely used them, whereas 94% (*n* = 16) were unfamiliar. The Cambridge Knee Injury Tool was unfamiliar to all participants.

**FIGURE 6:**
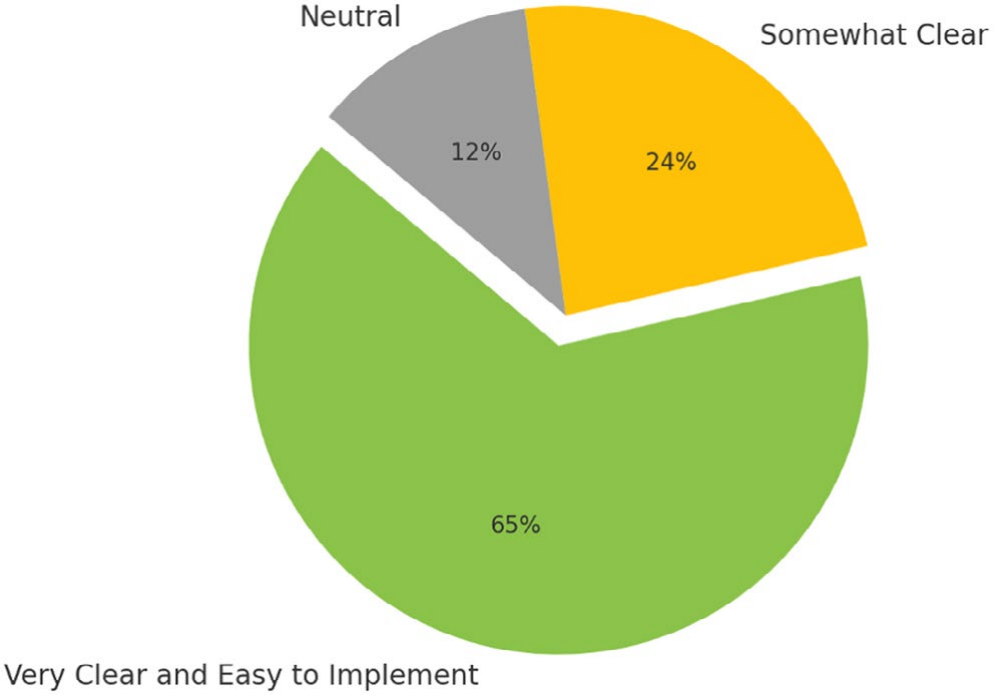
A pie chart showing clinicians' perceptions of how easy the pathway was to implement.

Regarding the proposed acute knee injury tool and pathway, a majority of respondents (77%, *n* = 13) found the tool very intuitive and easy to use, while 18% (*n* = 3) found it somewhat intuitive, and 6% (*n* = 1) found it somewhat difficult to use. Regarding the clarity of the clinical pathway, 65% (*n* = 11) found it very clear and easy to implement, 24% (*n* = 4) found it somewhat clear, and 12% (*n* = 2) were neutral (Figure 6).

Regarding the usefulness of risk stratification in guiding management, 53% (*n* = 9) felt it aligned well with their current clinical practice, while 47% (*n* = 8) believed it was mostly appropriate but might require some modifications. No respondents considered the system unsuitable. Finally, 71% (*n* = 12) thought the pathway would significantly streamline patient management in rural and resource‐limited settings, while 29% (*n* = 5) felt it would be somewhat useful.

## 4 Discussion

Implementing the Acute Knee Injury Pathway may lead to several important clinical and operational improvements in resource‐limited healthcare settings. The pathway integrates red and yellow flag screening criteria, the Ottawa Knee Rules, and the Cambridge Knee Injury Tool to provide a comprehensive guide for managing both bony and soft‐tissue knee injuries.

The pathway development aimed to support clinical decision‐making through the standardisation of care. In doing so, clinicians could have greater confidence in assessing and managing knee injuries using the pathway. This standardisation of clinical decision‐making may promote consistency in how patients are triaged and treated, reducing variability in care. This pathway also has the potential to enhance patient safety and optimise discharge processes. Patients with low‐risk injuries can be discharged safely with clear safety netting advice, reducing unnecessary follow‐up appointments and emergency department reattendance. Medium‐risk cases can receive clearer referral pathways, ensuring that patients who require specialist assessment are directed to the most appropriate service. High‐risk patients can be identified earlier, allowing faster access to imaging, specialist consultation, and potential surgical intervention. Faster access to specialist treatment has the potential to improve the care of high‐risk patients and may have an impact on lower SES rural populations, where timely care of STKIs may reduce the incidence of post‐traumatic osteoarthritis [7, 8].

Aligning with clinical guidelines, the pathway may increase efficiency in resource utilisation by helping to reduce unnecessary imaging. This may subsequently increase the chance of clinical uptake. This is supported by the results that demonstrate most surveyed rural clinicians found the pathway aligned well with existing guidelines and was consistent with best practices for managing acute knee injuries. Rural clinicians agreed that the risk stratification model provided a structured, evidence‐based framework that was easy to apply in a rural setting. Subsequent support for clinical uptake was strong, with rural doctors endorsing the pathway as a valuable tool for streamlining patient management.

Importantly, this pathway demonstrated its potential to address the significant barriers in rural healthcare delivery, including limited access to imaging, variable clinician experience in musculoskeletal assessment, and specialist availability. The structured approach may be particularly useful in settings where access to MRI is restricted, providing clear guidance on when referral for advanced imaging is necessary. In rural hospitals where telehealth services are available, clinicians valued the potential in integrating a non‐invasive screening tool into virtual fracture clinics or telehealth specialist consultations for enhanced patient care.

A limitation of this study is the lack of involvement of allied health professionals. This study focused exclusively on medical officers due to its modest scale and the need for internal consistency across respondents. Including allied health respondents in a small sample would have skewed representation and reduced comparability. This is because one of the main barriers to diagnosis is the lack of clinicians' experience and accuracy in assessing musculoskeletal injuries, which is a unique barrier that physiotherapists do not face to the same extent. Additionally, we note that in rural emergency departments, a medical officer should be involved in all initial patient presentations, hence the relevance of focusing on medical perspectives. Previous Delphi studies that informed this study have included a multidisciplinary cohort of allied health professionals (e.g., physiotherapists, emergency nurse practitioners) to identify themes of research priorities and diagnostic challenges in STKIs [5, 24].

There is a range of recommendations for further action to promote implementation and uptake. These include conducting ongoing training sessions for rural clinicians and allied health professionals to reinforce competency in knee assessment and improve confidence in pathway utilisation. Educating clinicians and allied health professionals on knee clinical decision tools would be a priority. Involving a wider range of healthcare stakeholders in this training would also improve uptake. Community and patient involvement would involve conducting educational outreach to patients regarding acute injury management, red flags, and appropriate healthcare‐seeking behaviours.

Future research would involve conducting a pilot study on the clinical integration of the pathway across rural hospitals and primary care centres to assess its effectiveness. This would include implementing longitudinal follow‐up of patients managed using the pathway to evaluate clinical outcomes such as return to function, missed diagnoses, and patient satisfaction. Further considerations involve exploring telemedicine options for specialist orthopaedic/sports medicine input in remote areas and advocating for funding to improve point‐of‐care access to imaging in rural hospitals.

## 5 Conclusion

The Acute Knee Injury Pathway was perceived by rural clinicians to have the potential to improve clinical decision‐making, resource efficiency, and patient safety, all while adhering to best practices. Clinician feedback was positive, with support for continued use and further expansion. By addressing diagnostic challenges in rural settings, the pathway has the potential to standardise knee injury management, reduce unnecessary imaging, and enhance patient outcomes while ensuring appropriate use of limited healthcare resources.

## Author Contributions

T.M: Conceptualisation, Methodology, Investigation, Formal analysis, Writing Original draft, Writing Review and Editing. B.G: Investigation, Writing Review and Editing. M.D: Investigation, Data Curation, Formal Analysis, Writing ‐ Review and Editing. S.M: Conceptualisation, Supervision, Guarantor.

## Ethics Statement

This study involved the analysis of anonymised survey data and did not involve any intervention or collecting identifiable personal information. As such, formal ethical approval was not required by institutional guidelines.

## Conflicts of Interest

The authors declare no conflicts of interest.

## Supporting information


**Appendix S1:** Survey: perspectives on acute knee injury assessment.

## Data Availability

The data that support the findings of this study are available from the corresponding author upon reasonable request.
