# Supplementary material for: Informing a Clinical Pathway for Acute Knee Injuries: Survey Insights From Rural Clinicians
Source: Aust J Rural Health. 2025 Aug 27;33(4):e70086. doi: 10.1111/ajr.70086 (PMC12381914; doi:10.1111/ajr.70086)
Supplement: Supplementary file 1 — Appendix S1: Survey: perspectives on acute knee injury assessment. [file AJR-33-0-s001.docx]

| Survey: Perspectives on Acute Knee Injury Assessment | | | | | |
| --- | --- | --- | --- | --- | --- |
| Section | | | **Question** | **Answer Options** | |
| PART 1 | | | | | |
| 1. Demographics & Clinical Experience | | | How many years of clinical experience do you have? | - <2 years - 2–5 years - 6–10 years - 11+ years | |
|  | | | How often do you assess patients with acute knee injuries? | - Daily - Weekly - Monthly - Rarely | |
| 2. Proficiency in Acute Knee Injury Assessment | | | How confident are you in assessing acute knee injuries? | - Very confident - Somewhat confident - Neutral - Somewhat unconfident - Very unconfident | |
|  | | | How confident are you in performing special tests for acute knee injuries? | - Very confident - Somewhat confident - Neutral - Somewhat unconfident - Very unconfident | |
|  | | | Which aspects of knee assessment do you find most challenging?  (Tick any) | - Pain and guarding during assessment - Effusion during assessment - Determining the need for imaging - Determining the need for specialist referral - Other (please specify): ________ | |
|  | | | How would you rate the current acute knee injury pathways at your institution? | - Very effective - Somewhat effective - Neutral - Somewhat ineffective - Very ineffective - Not aware of any | |
| 3. Knowledge of Clinical Imaging Guidelines | | | Are you familiar with the Ottawa Knee Rules for imaging guidance? | - Yes, very familiar and apply them regularly - Somewhat familiar but do not consistently apply them - Aware of them but rarely use them - Not familiar | |
|  | | | Are you familiar with the Pittsburgh Knee Rules for imaging guidance? | - Yes, very familiar and apply them regularly - Somewhat familiar but do not consistently apply them - Aware of them but rarely use them - Not familiar | |
|  | | | Are you familiar with the Cambridge Knee Injury Tool for referral guidance? | - Yes, very familiar and apply them regularly - Somewhat familiar but do not consistently apply them - Aware of them but rarely use them - Not familiar | |
|  | | | In your experience, how strictly are clinical imaging guidelines followed in your hospital? | - Always followed - Often followed but with some exceptions - Rarely followed, imaging is overused - Rarely followed, imaging is underused | |
| 4. Barriers to Knee Injury Assessment in Rural & Remote Settings | | | What are the biggest barriers to assessing acute knee injuries in rural areas? (Tick any) | - Limited access to imaging (X-ray, MRI) - Lack of specialist consultation (orthopaedics, sports medicine) - Difficulty in arranging follow-up care - Limited training or confidence in knee assessment - Patient reluctance to travel for specialist care - Other (please specify): ________ | |
|  | | | In rural settings, how often do you feel pressured to refer patients for imaging or specialist consultation due to diagnostic uncertainty? | - Very often - Sometimes - Rarely - Never | |
| PART 2 | | | | | |
| Section 5: Tool Perception & Usability | | | How would you rate the usability of the questionnaire? | - Very intuitive and easy to use - Somewhat intuitive, but could be improved - Neutral - Somewhat difficult to use - Very difficult to use | |
|  | | | How clear and practical do you find the proposed acute knee management pathway for acute knee injuries? | - Very clear and easy to implement - Somewhat clear but could be improved - Neutral - Somewhat unclear and difficult to implement - Very unclear | |
|  | | | Do you think the risk stratification (Low, Medium, High risk) appropriately guides patient management? | - Yes, it aligns well with current clinical practice - Mostly, but some modifications are needed - Neutral - Not really, adjustments would be required - No, it does not fit into my current practice | |
|  | | | In rural or resource-limited settings, do you think the pathway would help streamline patient management? | - Yes, significantly - Somewhat - Neutral - Not really - No, it would not be useful | |
|  | **Any other thoughts or comments:** | | | [Text entry] | |
